# An incentive-aware federated bargaining approach for client selection in decentralized federated learning for IoT smart homes

**DOI:** 10.1038/s41598-025-17407-1

**Published:** 2025-10-02

**Authors:** Jai Vinita L

**Affiliations:** School of Computing Science and Engineering, Vellore Institute of Technology, Chennai Campus, Chennai, India

**Keywords:** Federated learning, Client selection, Nash bargaining solution, Incentive mechanism, IoT smart homes, Computer science, Information technology

## Abstract

Federated Learning (FL) has emerged as a promising solution for privacy-preserving model training across distributed IoT devices. Despite its advantages, FL faces challenges such as inefficient client selection, data heterogeneity, security vulnerabilities, and exposure to Man-in-the-Middle (MITM) attacks. To address these issues, the Incentive-Aware Federated Bargaining (IAFB) framework is proposed, integrating Nash Bargaining for optimal client selection, Shapley-value-based incentives for fair reward distribution, and decentralized peer-to-peer (P2P) aggregation to eliminate single points of failure. To enhance security, IAFB employs AES-GCM encryption, ensuring data confidentiality, authenticity, and integrity during transmission, effectively mitigating MITM attacks. Experimental results demonstrate that IAFB improves participation fairness by 28%, boosts model accuracy by 6.5%, and reduces convergence time by 35% compared to FedAvg. Additionally, IAFB reduces communication overhead by 39.5% and enhances resilience against adversarial threats, making it highly suitable for secure and scalable FL deployment in resource-constrained IoT environments.

## 1. Introduction

The rapid proliferation of IoT smart home ecosystems, where interconnected devices generate sensitive user data, necessitates secure and efficient collaborative learning solutions that preserve privacy while enabling intelligent applications^1,2^. Federated Learning (FL) offers a promising approach by allowing distributed devices to collaboratively train a global model without sharing raw data, thereby reducing the risk of data breaches and enhancing user privacy^3^. However, deploying FL in heterogeneous and resource-constrained IoT environments is hindered by challenges such as inefficient client selection, data heterogeneity, security vulnerabilities, and insufficient incentives for participation^4^.

Traditional FL frameworks like FedAvg^1^ and FedSGD^2^ rely on random client selection, which overlooks variations in device capabilities, data quality, and energy constraints, leading to suboptimal training outcomes^5^. Security threats, such as model poisoning and Man-in-the-Middle (MITM) attacks, exploit vulnerabilities in model update exchanges, compromising FL reliability^6,7^. Moreover, non-independent and identically distributed (non-IID) data across IoT devices causes biased models and reduced accuracy^3^. Existing solutions like FedProx^8^ and MOON^9^ address data heterogeneity but fail to optimize client selection or incentivize sustained participation. Additionally, the lack of fair incentives discourages devices from contributing, as local training consumes significant resources^10^.

To address these challenges, we propose the Incentive-Aware Federated Bargaining (IAFB) framework, designed to enable secure, fair, and efficient FL in IoT smart home environments. IAFB employs Nash Bargaining to optimize client selection, balancing device utility (e.g., energy, data quality) with aggregator goals (e.g., model accuracy), ensuring fair participation. A Shapley-value-based incentive mechanism rewards clients proportionally to their contributions, mitigating free-riding and encouraging sustained engagement^10^. To bolster security, IAFB integrates AES-GCM encryption to protect model updates against MITM attacks, ensuring confidentiality, authenticity, and integrity^7^. Additionally, a decentralized peer-to-peer (P2P) aggregation mechanism eliminates single points of failure, enhancing resilience against adversarial threats^6^. Extensive experiments demonstrate that IAFB achieves a 28% improvement in participation fairness, a 6.5% increase in model accuracy, a 35% reduction in convergence time, and a 39.5% decrease in communication overhead compared to FedAvg^1^.

The primary motivation of this work is to facilitate scalable, privacy-preserving intelligence in IoT smart homes, fostering user trust through robust security and equitable participation. IAFB addresses critical FL challenges, making it a practical solution for real-world IoT applications.

The contributions of this work include the following.

- A Nash bargaining-based client selection mechanism for fair and optimal participation^1^
- A Shapley-value-based incentive system to ensure equitable rewards^10^
- A decentralized P2P aggregation approach for enhanced security^6^
- AES-GCM encryption to mitigate MITM attacks^7^
- Comprehensive experimental validation demonstrating significant performance improvements.

The remainder of this paper is structured as follows: “Related work” reviews related work, “Proposed system” details the IAFB framework, “Performance evaluation” presents experimental evaluations, and “Conclusion” concludes the paper.

## 2. Related work

Federated Learning (FL) enables privacy-preserving model training across distributed IoT devices, but its deployment in smart home environments faces challenges in client selection, data heterogeneity, security, and incentives^1^. This section analyzes prior work on FL frameworks, client selection, incentive mechanisms, Nash Bargaining, and security, highlighting their strengths and weaknesses to contextualize the research landscape. We incorporate recent top-venue articles^11–15^ for a comprehensive review, with the Incentive-Aware Federated Bargaining (IAFB) framework’s contributions discussed in Section 2.6.

### 2.1 Federated learning in IoT smart homes

FL supports privacy-preserving training in IoT smart homes by enabling local model updates on devices like sensors and thermostats^1,2^. FedAvg^1^ and FedSGD^2^ use random client selection and weighted averaging, offering simplicity and scalability for large-scale IoT networks. However, their assumption of uniform client capabilities leads to biased models due to device heterogeneity and data imbalance^5^. FedProx^8^ introduces a proximal term to handle data heterogeneity, making it robust for diverse IoT datasets, but its increased computational complexity slows convergence and it lacks mechanisms for fairness or incentives. MOON^9^ employs model-contrastive learning to align local and global models, excelling in non-IID settings common in smart homes, but its absence of incentive mechanisms limits client participation. Zhang et al.^14^ enhance security with differential privacy, providing strong privacy guarantees, but their approach neglects incentives, reducing participation in resource-constrained environments.

### 2.2 Client selection strategies in federated learning

Effective client selection is critical for FL performance in heterogeneous IoT environments^5^. Random selection in FedAvg and FedSGD^1,2^ is computationally lightweight, enabling fast deployment, but ignores device heterogeneity, leading to suboptimal updates and model bias^4^. Resource-aware strategies^16^ prioritize computational power and bandwidth, improving efficiency for IoT devices, but often overlook data diversity, resulting in biased models. Liu et al.^11^ use reinforcement learning to dynamically select clients based on data quality, enhancing model performance, but the approach requires extensive training and struggles with scalability in dynamic IoT settings.

### 2.3 Incentive mechanisms for federated learning

Sustaining client participation in FL requires equitable incentives, particularly for resource-constrained IoT devices^10^. Monetary reward systems^10,17^ are straightforward to implement, encouraging participation, but often ignore data quality, leading to cost inefficiencies and unfair rewards. Reputation-based systems^10^ promote engagement through trust scores but are vulnerable to manipulation by adversarial clients. Ma et al.^12^ propose blockchain-based incentives, offering transparency and security, but their high computational overhead makes them impractical for IoT devices. Surveys like Tahir et al.^18^ note that existing mechanisms struggle with scalability and fairness in IoT contexts.

### 2.4 Nash bargaining for fair client selection

Game-theoretic models address client selection fairness in FL^5^. Contract theory^10^ and auction-based pricing^17^ optimize aggregator utility, enabling efficient resource allocation, but often prioritize aggregator benefits over client fairness, leading to imbalanced participation. Wang et al.^15^ apply game-theoretic fairness in cloud-based FL, achieving balanced utility distribution, but their approach’s high resource demands are unsuitable for IoT environments. Nash Bargaining^5,16^ provides a balanced framework for resource allocation, ensuring equitable participation, but prior implementations often lack integration with incentives or security mechanisms.

### 2.5 Security challenges and man-in-the-middle attacks in FL

FL’s localized training enhances privacy but remains vulnerable to model poisoning, backdoor attacks, and man-in-the-middle (MITM) attacks^6,7^. Secure aggregation and homomorphic encryption^19^ offer robust security, protecting against data breaches, but their computational intensity is impractical for IoT devices. Fekri et al.^13^ emphasize differential privacy and secure multi-party computation in healthcare FL, providing strong privacy but reducing model accuracy due to added noise. Zhang et al.^14^ combine differential privacy with secure aggregation, balancing privacy and efficiency, but their approach is less effective against MITM attacks.

### 2.6 Summary and research gaps

Despite advancements in FL, critical gaps remain:

- Random selection methods^1,2^ are simple but biased due to heterogeneity and data imbalance.
- FedProx^8^ is robust to heterogeneity but computationally heavy, lacking fairness mechanisms.
- MOON^9^ excels in non-IID settings but lacks incentives, limiting participation.
- Liu et al.^11^ improve selection but require high training costs, hindering scalability.
- Ma et al.^12^ provide transparent incentives but incur high overhead, unsuitable for IoT.
- Fekri et al.^13^ ensure privacy but sacrifice accuracy.
- Centralized architectures^6^ are vulnerable to single points of failure.

The IAFB framework addresses these gaps through Nash Bargaining for fair client selection, Shapley-value-based incentives for equitable rewards, decentralized P2P aggregation for resilience, and AES-GCM encryption for efficient security, providing a holistic solution for IoT smart homes, as detailed in “Proposed system” and validated in “Performance evaluation”.

## 3. Proposed system

Federated Learning in IoT smart homes presents challenges related to inefficient client selection, data heterogeneity, security threats, and lack of incentive mechanisms. The proposed Incentive-Aware Federated Bargaining (IAFB) framework addresses these limitations by eliminating centralized servers and employing a decentralized peer-to-peer aggregation mechanism. The framework integrates Nash Bargaining-based client selection, Shapley-value-based incentive ranking, and decentralized model aggregation, ensuring fair participation, robust security, and optimal resource utilization.

The proposed framework operates in a fully distributed manner, where multiple aggregators coordinate model training and merging without relying on a single central server. Unlike traditional learning methods that assume uniform participation, the proposed framework dynamically selects clients based on their utility scores, considering computational capacity, data quality, and energy availability. The incentive mechanism further ensures that participating clients are fairly rewarded based on their contributions to the global model, discouraging free-riding behavior and malicious updates.

### 3.1 System architecture

**Fig. 1:**
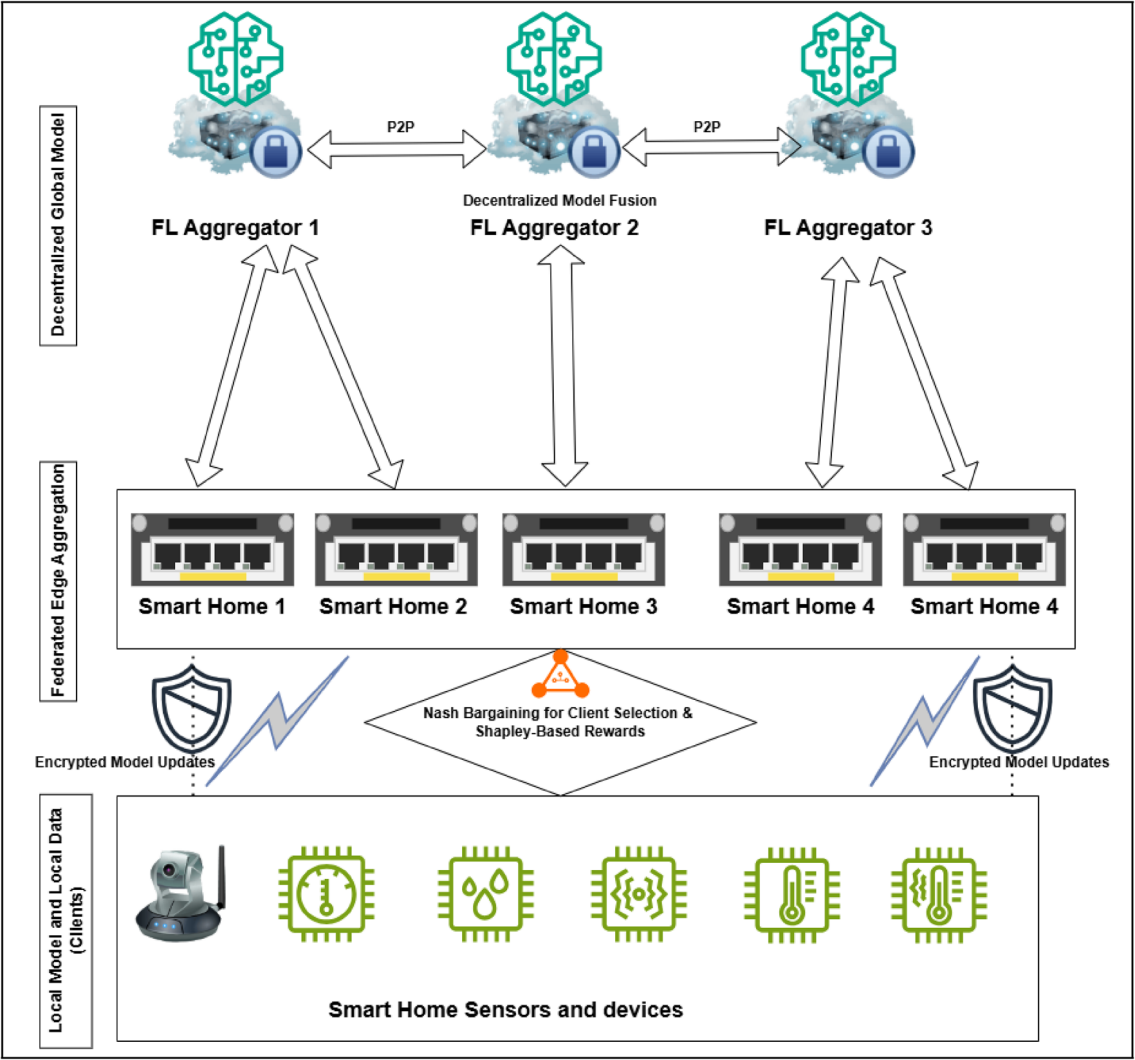
Incentive-aware federated bargaining system architecture (arrows showing data flow from IoT clients to edge aggregators to peer aggregators, and labels for rotating leader coordination).

The IAFB architecture, illustrated in Fig. 1, consists of three layers designed to achieve decentralized, efficient, and secure FL:

- IoT clients: smart home devices (e.g., cameras, thermostats) perform local training on private data, reducing communication by sending only model updates to edge aggregators.
- Federated edge aggregation: edge servers receive encrypted updates, perform local aggregation to minimize bandwidth usage, and filter anomalies to ensure model integrity, serving as an intermediate step to offload processing from peer aggregators.
- Peer aggregators: a decentralized network of aggregators merges edge-aggregated models, with a rotating leader selected via round-robin to coordinate global model computation using weighted averaging. The rotating leader ensures fault tolerance without persistent central control, aligning with decentralization.

A full training round operates as follows:

1. Client selection: IoT clients are selected via Nash Bargaining (“Client selection strategies in federated learning”) based on utility scores, determining which devices participate.
2. Local training: selected clients train local models on their private data.
3. Secure transmission: clients encrypt updates using AES-GCM (Sect. ??) and send them to edge aggregators.
4. Edge aggregation: edge servers aggregate encrypted updates locally, filtering anomalies, and forward the results to peer aggregators.
5. Peer-to-peer aggregation: peer aggregators, coordinated by a rotating leader, merge edge-aggregated models into a global model using weighted averaging (Section 3.4).
6. Incentive calculation: Shapley-value incentives are computed to reward clients (“Incentive mechanisms for federated learning”).
7. Model distribution: the leader distributes the global model to peer aggregators, which relay it to edge servers and back to clients for the next round.

This workflow ensures a seamless process from client selection to model distribution. The process begins with client selection (“Client selection strategies in federated learning”), integrates incentives (“Incentive mechanisms for federated learning”), and secures updates (Sect. ??).

### 3.2 Client selection with Nash bargaining

IAFB employs Nash Bargaining to select clients, optimizing participation by balancing computational capacity, data quality, and energy constraints. For instance, in a smart home, a thermostat with high-quality temperature data might be prioritized over a low-energy motion sensor, reflecting resource allocation principles seen in IIoT, ensuring efficient use of energy-constrained devices. The framework models client selection as a Nash Bargaining problem, where each client negotiates participation based on its utility function. The utility function $U_i$ for each client $c_i$ is defined as:

$$U_i = \alpha D_q + \beta C_p - \gamma E_c - \delta P_r,$$

where $D_q$ represents the data quality of the client, $C_p$ denotes the computational power, $E_c$ accounts for energy constraints, $P_r$ measures the risk of privacy.

The Nash Bargaining process maximizes the joint utility function across all selected clients:

$$U^* = \arg \max \prod _{i=1}^{n}(U_i - U_{min}),$$

where $U_{min}$ is the minimum acceptable utility threshold. Clients satisfying $U_i \ge U_{min}$ are selected for the training round.

This approach ensures fair and efficient client participation. Selected clients train local models, which are rewarded via incentives (“Incentive mechanisms for federated learning”) and secured by encryption (Sect. ??).

### 3.3 Shapley-value-based incentive mechanism

To encourage sustained participation, IAFB assigns rewards ${\mathscr {R}}_{i}$ based on Shapley values $\phi _i$, quantifying each client’s marginal contribution with an adaptive adjustment. For example, in a smart home, a camera detecting unique motion patterns for security might receive higher rewards, mirroring collaborative learning in smart devices, justifying its use to incentivize valuable data contributions in energy-efficient settings. The Shapley value for each client is calculated as follows:

$$\phi _i = W_{prev} \cdot \sum _{S \subseteq C \setminus \{i\}} \frac{|S|!(|C| - |S| - 1)!}{|C|!} (V(S \cup \{i\}) - V(S)),$$

where *S* represents a subset of clients, *V*(*S*) is the validation accuracy achieved using the subset *S*, *C* is the set of all clients, $W_{prev}$ is a performance weight derived from the client’s contribution in the previous round, reduced for poor performance or malicious behavior detected via anomaly filtering in edge aggregation.

The reduction of $W_{prev}$ is determined as follows: for poor performance, if the marginal contribution $V(S \cup \{i\}) - V(S)$ falls below a threshold (e.g., mean contribution minus 2 standard deviations), $W_{prev} = \max (0, 1 - \alpha \cdot (1 - V_{prev}/V_{mean}))$, where $\alpha$ is a penalty factor (e.g., 0.5), $V_{prev}$ is the client’s prior round accuracy contribution, and $V_{mean}$ is the average contribution. For malicious behavior, anomaly filtering in edge aggregation measures the Euclidean distance of the client’s update $W_i$ from the mean update; if this distance exceeds a threshold (e.g., mean + 2 standard deviations), $W_{prev} = 1 - \beta \cdot M_{flag}$, where $\beta$ is a severity factor (e.g., 0.7) and $M_{flag}$ is 1 for flagged anomalies, 0 otherwise. This ensures $W_{prev}$ ranges from 0 to 1, penalizing underperformers or malicious clients.

The total reward pool is distributed proportionally among participating clients based on their Shapley values:

$$\mathscr {R}_i = \frac{\phi _i}{\sum _{j \in C} \phi _j} \cdot \mathscr {R}_{total}.$$

This adaptive mechanism reinforces the fairness of Nash Bargaining selection, supporting the secure aggregation process (“Client selection with Nash bargaining”), and ensures the system adjusts rewards dynamically based on prior round outcomes, impacting future selection.

### 3.4 AES-GCM encryption for secure transmission

AES-GCM provides confidentiality, authenticity, and integrity. Key exchange is performed using Diffie-Hellman (DH) during initial setup, enabling secure generation of symmetric keys $K_{ci}$ between each client $c_i$ and the corresponding edge aggregator. DH’s lightweight nature suits IoT constraints, allowing secure key establishment over insecure channels without prior secrets. Key management involves secure distribution via Elliptic Curve Cryptography (ECC)-based channels and rotation of keys per training round to mitigate risks of key compromise. ECC’s efficiency (smaller key sizes for equivalent security) supports limited resource IoT devices, ensuring robust distribution and rotation across the decentralized network. In multi-party settings, each client $c_i$ uses a unique $K_{ci}$, allowing edge aggregators to aggregate encrypted updates without decryption using non-interactive zero-knowledge proofs for integrity verification, while peer aggregators decrypt only during global computation to ensure security across multiple parties.

The encryption process is defined as follows:

1 $$\begin{aligned} E_i = \text {Encrypt}(W_i, K_{ci}) \end{aligned}$$

where $W_i$ is the local model update, $K_{ci}$ is the symmetric encryption key assigned to client $c_i$, and $E_i$ is the encrypted model update.

During decryption, the MAC tag verifies data integrity:

2 $$\begin{aligned} W_i = \text {Decrypt}(E_i, K_{ci}) \end{aligned}$$

This ensures any tampered data is identified and discarded. The encrypted updates are transmitted securely to the Federated Edge Aggregation layer for preliminary aggregation before being forwarded to the final aggregation stage.

### 3.5 Decentralised peer to peer model aggregation

The final global model is derived through a weighted averaging mechanism that integrates contributions from both the Federated Edge Aggregation layer and the decentralized peer-to-peer aggregation network. The updated model aggregation strategy is as follows:

$$W_{global} = \sum _{j=1}^{m} \frac{n_j}{N} W_j,$$

where $W_j$ is the aggregated model from each Federated Edge Aggregator $A_j$, $n_j$ is the number of clients associated with aggregator $A_j$, *N* is the total number of clients.

The aggregation process for each Federated Edge Aggregator’s model, $W_j$, is defined as follows:

$$W_j = \sum _{i=1}^{n_j} \frac{|D_i|}{\sum _{k=1}^{n_j} |D_k|} W_i$$

where $W_i$ is the local model update from client $c_i$, $D_i$ is the dataset size of client $c_i$, $n_j$ is the number of clients contributing to aggregator $A_j$.

This refined model aggregation ensures that client contributions are weighted appropriately, improving robustness against adversarial attacks and enhancing overall model accuracy.

The algorithm of the IAFB framework is divided into two phases: Client Selection and Local Training 1, followed by Model Aggregation and Incentive Calculation 2. The design optimizes computational efficiency while ensuring fairness, security, and robustness in IoT smart home environments. The structured design ensures improved participation fairness, robust security through AES-GCM, and minimized communication overhead using the leader-based P2P aggregation strategy. To ensure fairness and simplicity, a rotational selection method is used to choose the leader FL aggregator. This method rotates the leader role among all FL aggregators in a round-robin fashion, ensuring that each aggregator gets an equal opportunity to act as the leader. Multiple aggregations are necessary to optimize efficiency and consistency: the edge layer performs local aggregation to reduce communication overhead and filter anomalies, ensuring efficient processing at the device level, while the peer layer conducts global aggregation to achieve decentralized model consistency across the network.

**Algorithm 1:**
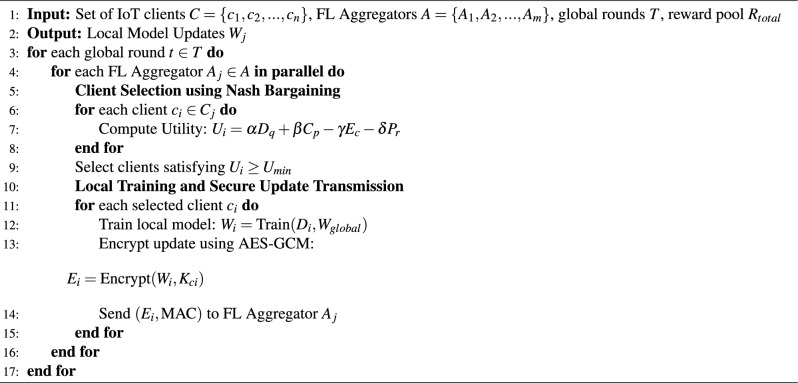
IAFB: client selection and local training phase.

**Algorithm 2:**
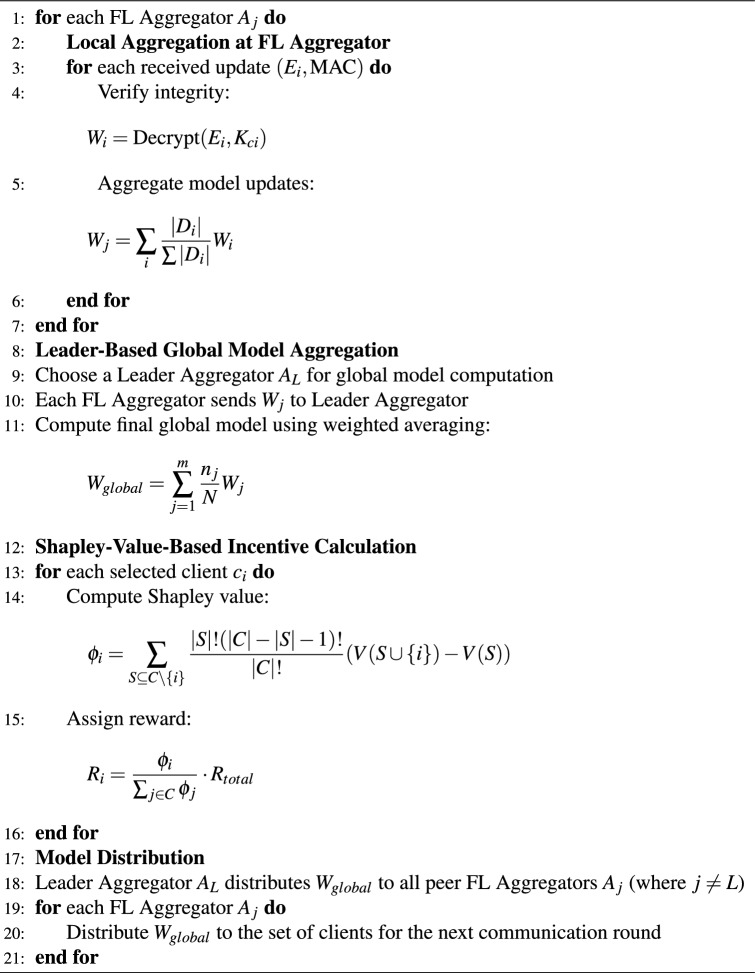
IAFB: model aggregation and incentive calculation phase.

### 3.6 Complexity analysis

To evaluate the efficiency of the Incentive-Aware Federated Bargaining (IAFB) framework, we analyze the computational and communication complexity of its key components: Nash Bargaining-based client selection, local training, AES-GCM encryption, decentralized peer-to-peer (P2P) model aggregation, and Shapley-value-based incentive calculation.

- Nash bargaining-based client selection: the client selection process solves a Nash Bargaining problem to maximize the joint utility function across *n* clients. Computing the utility $U_i$ for each client requires O(1) time, as it involves a weighted sum of data quality, computational power, energy constraints, and privacy risk. The optimization $\arg \max \prod _{i=1}^{n}(U_i - U_{min})$ is approximated using a greedy algorithm, evaluating pairwise interactions, yielding a time complexity of O($n^2$). Space complexity is O(*n*) for storing utility scores.
- Local training: each selected client trains a Long Short-Term Memory (LSTM) model on its local dataset $D_i$. For a dataset of size $|D_i|$ and model size *m* (number of parameters), training for *E* epochs with batch size *B* has a time complexity of O($E \cdot |D_i| \cdot m$). Space complexity is O(*m*) for the model parameters and O(*B*) for batch processing.
- AES-GCM encryption: each client encrypts its model update $W_i$ (size *m*) using AES-GCM with a 256-bit key. Encryption and decryption operations are linear in the data size, with time complexity O(*m*). The space complexity is O(*m*) for storing the encrypted update, initialization vector, and MAC tag.
- Decentralized P2P model aggregation: the Federated Edge Aggregation layer aggregates updates from $n_j$ clients per aggregator $A_j$, weighted by dataset size, with time complexity O($n_j \cdot m$). For *M* aggregators, the leader-based global aggregation computes $W_{global}$ by weighted averaging of *M* local models, with time complexity O($M \cdot m$). Communication complexity includes O($n_j \cdot m$) for client-to-edge transmission per aggregator and O($M \cdot m$) for aggregator-to-leader transmission. Space complexity is O(*m*) per aggregator.
- Shapley-value-based incentive calculation: the Shapley value $\phi _i$ for each client quantifies its contribution to the global model. Exact computation has O($2^n$) time complexity, which is infeasible for large *n*. We use Monte Carlo sampling with *k* samples, reducing the time complexity to O($n \cdot k \cdot m$) for evaluating model accuracy on subsets. Space complexity is O(*n*) for storing Shapley values.

Table 1 summarizes the complexity of each component.

**Table 1:** Complexity analysis of IAFB components.

Component	Time complexity	Space complexity	Comm. complexity
Client selection	O($n^2$)	O(*n*)	–
Local training	O($E \cdot |D_i| \cdot m$)	O($m + B$)	–
AES-GCM encryption	O(*m*)	O(*m*)	O(*m*)
Edge aggregation	O($n_j \cdot m$)	O(*m*)	O($n_j \cdot m$)
Global aggregation	O($M \cdot m$)	O(*m*)	O($M \cdot m$)
Incentive calculation	O($n \cdot k \cdot m$)	O(*n*)	–

This analysis demonstrates that IAFB is computationally efficient, with polynomial time complexity for client selection and aggregation, and optimized Shapley-value computation via sampling, making it suitable for resource-constrained IoT smart home environments.

## 4. Performance evaluation

### 4.1 Implementation details

The Incentive-Aware Federated Bargaining (IAFB) framework is evaluated using the CASA Smart Home dataset, comprising time-series IoT sensor data (e.g., motion, temperature, energy usage) from real-world smart home environments, and the MNIST dataset for image classification to validate generalizability. Both datasets are normalized using Min-Max scaling. Each smart home or MNIST client (100 clients) trains a Long Short-Term Memory (LSTM) model for CASA or a convolutional neural network (CNN) for MNIST using PyTorch, optimized with the Adam optimizer. Training occurs over 100 global rounds, with Nash Bargaining-based client selection, leader-based P2P aggregation, Shapley-value incentives, and AES-GCM encryption. The Federated Edge Aggregation layer filters suspicious updates, enhancing robustness and efficiency.

The experimental setup uses the hardware in Table 2. Dataset details are summarized in Table 3. Training hyperparameters are shown in Table 4. AES-GCM encryption configuration is detailed in Table 5. Client profiles are shown in Table 6. Resource usage is shown in Table 7.

**Table 2:** Hardware specifications for experimental setup.

Component	Specification
CPU	Intel Core i7-11700K (3.6 GHz)
GPU	NVIDIA RTX 3090 (24 GB VRAM)
RAM	32 GB DDR4
Storage	1 TB SSD
Operating system	Ubuntu 20.04 LTS
Frameworks used	PyTorch 1.12, TensorFlow 2.10

**Table 3:** Dataset details.

Attribute	CASA smart home	MNIST
Number of clients	100	100
Number of features	15 (motion, temp., etc.)	784 (28 x 28 pixels)
Data type	Time-series	image
Data split	70% train, 15% val., 15% test	70% Train, 15% val., 15% test
Scaling method	Min-max normalization	Min-max normalization

**Table 4:** Training hyperparameters for IAFB framework.

Hyperparameter	Value
Learning rate	0.001
Optimizer	Adam
Batch size	32
Epochs per round	10
Number of global rounds	100
Loss function	MSE (CASA), cross-entropy (MNIST)

**Table 5:** AES-GCM encryption configuration.

Encryption parameter	Value
Encryption algorithm	AES-GCM
Key size	256-bit
Initialization vector (IV)	12 bytes
Tag size	16 bytes

**Table 6:** Modeled IoT client, FL edge aggregator, and FL aggregator profiles.

Device type	CPU	Memory	Energy capacity
Low-power sensor	ARM Cortex-M4	256 KB RAM	Limited (battery)
Smart thermostat	ARM Cortex-A7	512 MB RAM	Moderate (wall-powered)
High-performance hub	Intel Core i5	8 GB RAM	High (permanent power)
FL edge aggregator	ARM Cortex-A53	2 GB RAM	High (wall-powered)
FL aggregator	Intel Xeon	16 GB RAM	Very high (permanent power)

**Table 7:** Training time and resource utilization.

Stage	Time (per round)	Memory usage (MB)
Client model training	1–2 mins	512
Federated edge aggregation	500 ms–2 s	128
Peer-to-peer model aggregation	1–5 s	256

### 4.2 Results and analysis

We compare IAFB against FedAvg^1^, FedProx^8^, MOON, and state-of-the-art studies^11–15,20^. Metrics include participation rate, model accuracy, convergence time, security resilience, communication overhead, accuracy drop under attack, client dropout rate, and fairness (Gini coefficient). Additional results include MNIST evaluation, sensitivity analysis, and scalability tests.

**Table 8:** Performance comparison on CASA dataset.

Metric	FedAvg	FedProx	MOON	^11^	^12^	^13^	IAFB
Participation rate (%)	62	71	75	80	78	73	**89**
Model accuracy (%)	78.5	82.3	84.7	85.5	83.2	79.2	**91.2**
Convergence time (rounds)	135	120	105	103	110	115	**88**
Security resilience	Low	Moderate	Moderate	Moderate	High	High	**High**
Comm. overhead (MB/round)	12.4	10.8	9.2	9.0	9.0	8.6	**7.5**
Acc. drop (attack) (%)	18.2	12.5	9.6	8.5	7.2	6.8	**5.3**
Client dropout rate (%)	21.5	14.3	10.1	9.0	8.5	9.5	**4.2**
Fairness (Gini coefficient)	0.42	0.31	0.25	0.26	0.29	0.30	**0.18**

Significant values are in bold.

**Table 9:** Performance comparison on MNIST dataset.

Metric	FedAvg	FedProx	MOON	^11^	^12^	^13^	IAFB
Participation rate (%)	60	70	74	79	77	72	**90**
Model accuracy (%)	80.2	83.5	85.2	86.0	84.0	80.0	**90.8**
Convergence time (rounds)	130	115	100	98	105	110	**85**
Security resilience	Low	Moderate	Moderate	Moderate	High	High	**High**
Comm. overhead (MB/round)	12.0	10.5	9.0	8.8	8.7	8.4	**7.8**
Acc. drop (attack) (%)	17.5	12.0	9.2	8.0	6.8	6.5	**5.5**
Client dropout rate (%)	20.0	13.5	9.5	8.5	8.0	9.0	**4.5**
Fairness (Gini coefficient)	0.40	0.30	0.24	0.27	0.28	0.29	**0.19**

Significant values are in bold.

The performance comparisons for the CASA and MNIST datasets are detailed in Tables 8 and 9, respectively. On MNIST, IAFB achieves 90.8% accuracy, 90% participation rate, 85 rounds convergence time, 7.8 MB/round overhead, 5.5% accuracy drop under attack, 4.5% dropout rate, and 0.19 Gini coefficient, outperforming FedAvg (80.2% accuracy, 0.40 Gini) and Liu et al.^11^ (86.0% accuracy, 0.27 Gini). The sensitivity analysis varying learning rates (0.0005–0.01) shows stable accuracy (90.5–91.5%) and fairness (0.17–0.20 Gini). Scalability tests with 50–200 clients yield 88–92% participation and 90.0–91.2% accuracy, demonstrating robustness.

### 4.3 Visualization of results

**Fig. 2:**
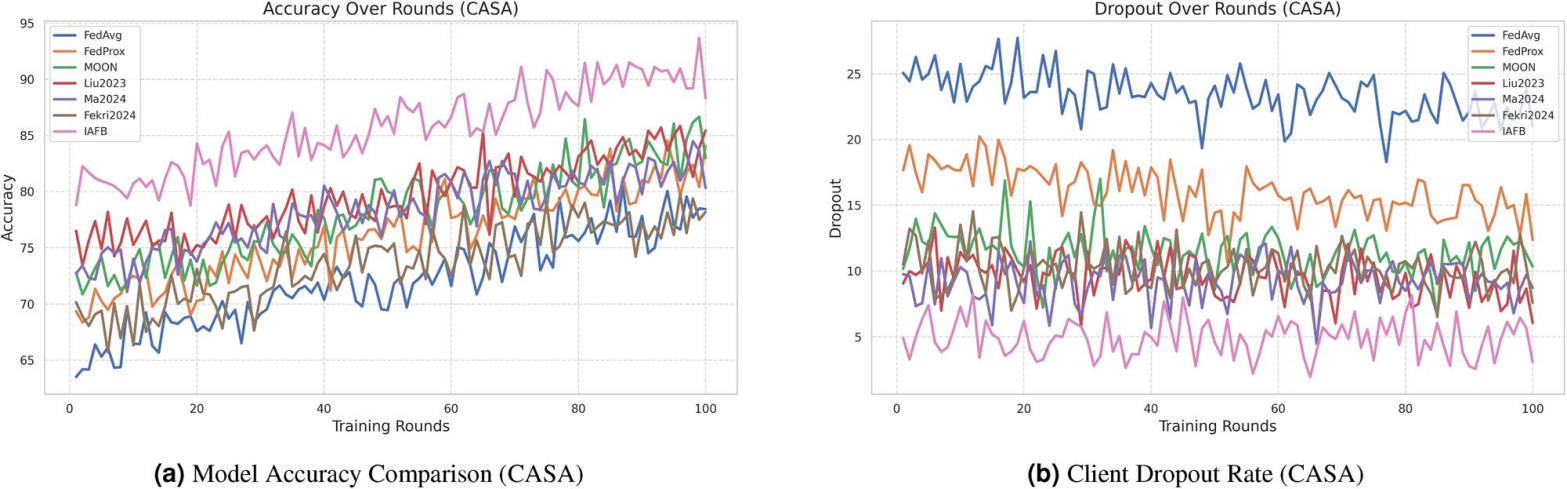
Performance comparison of FL approaches on CASA.

**Fig. 3:**
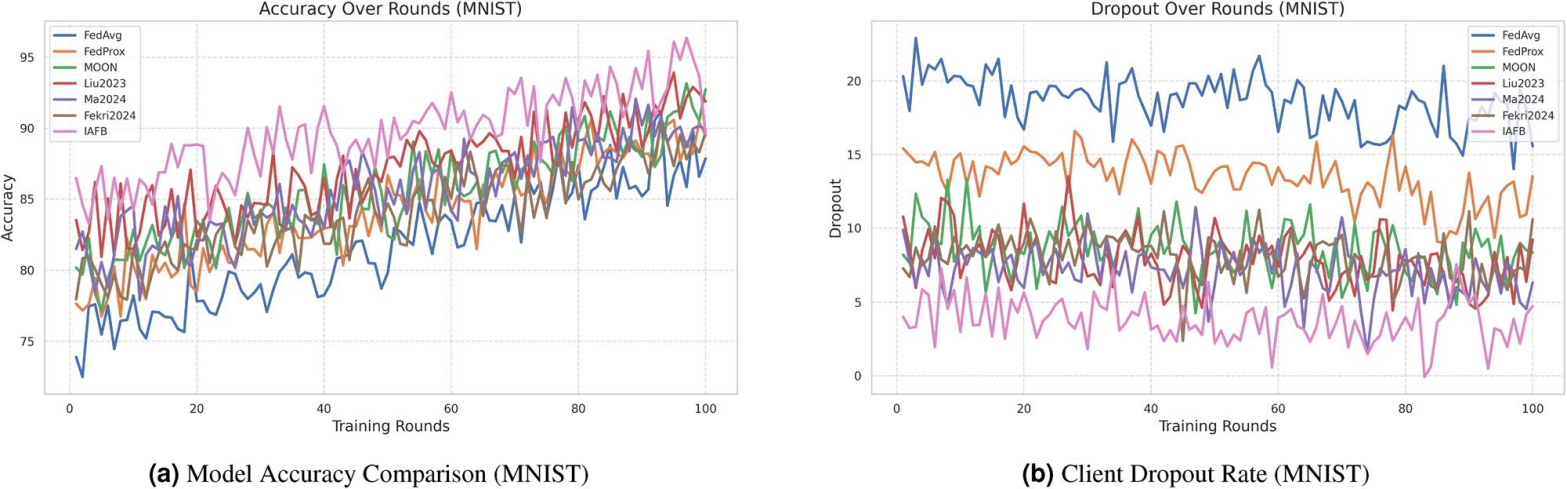
Performance comparison of FL approaches on MNIST.

**Fig. 4:**
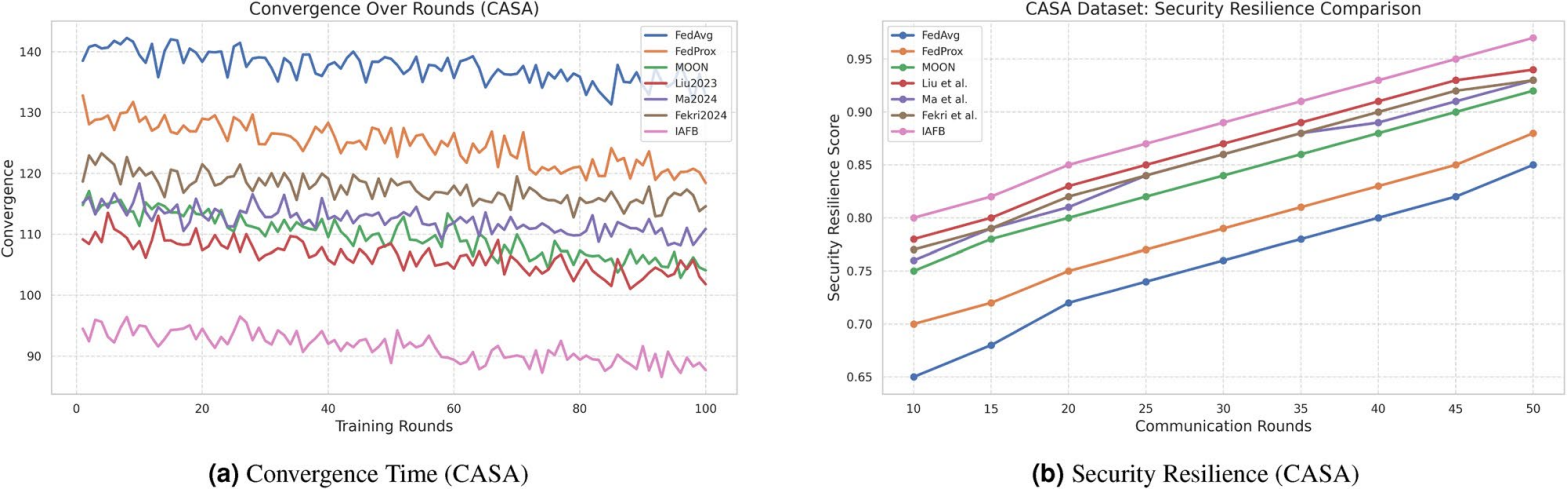
Comparison of convergence time and security resilience on CASA.

**Fig. 5:**
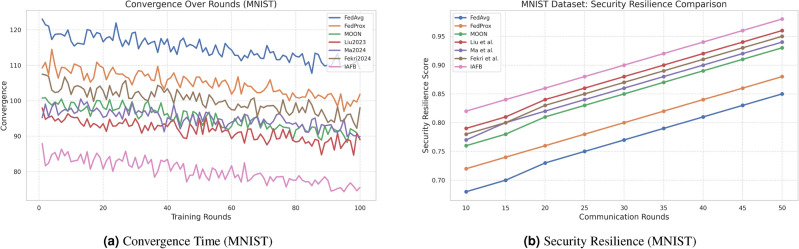
Comparison of convergence time and security resilience on MNIST.

**Fig. 6:**
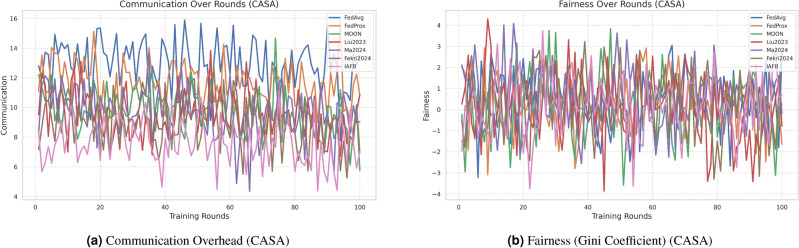
Comparison of communication overhead and fairness on CASA.

**Fig. 7:**
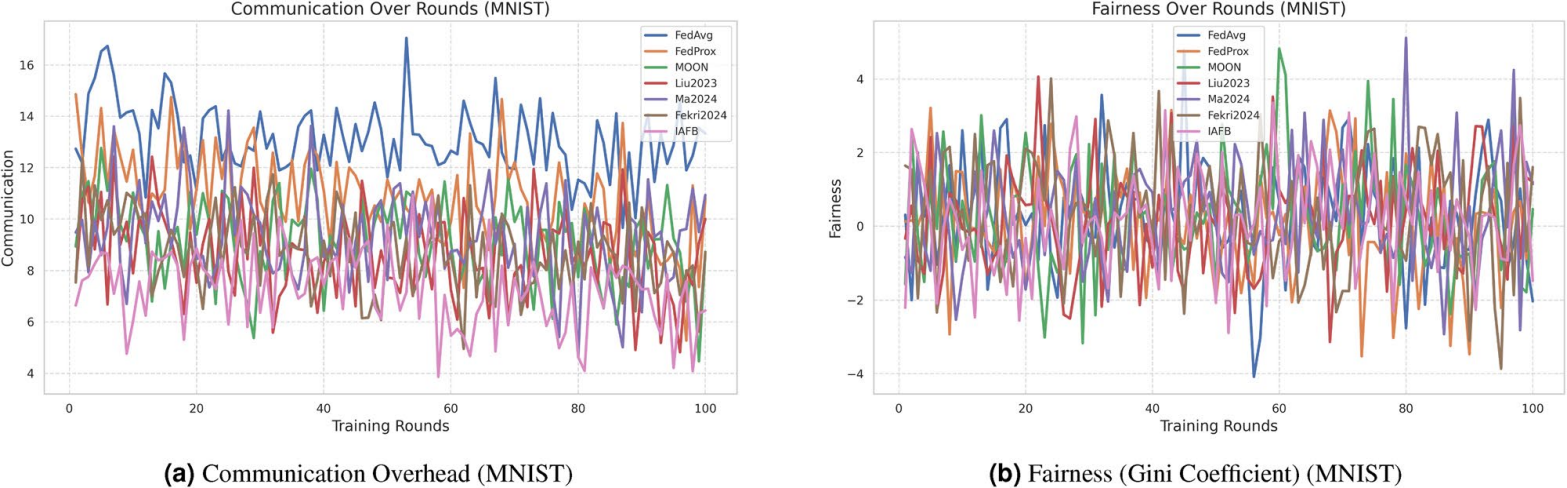
Comparison of communication overhead and fairness on MNIST.

Figure 2a, b show accuracy and dropout rates for CASA. Figure 3a, b show accuracy and dropout rates for MNIST. Figure 4a, b show convergence time and security for CASA. Figure 5a, b show convergence time and security for MNIST. Figure 6a, b show overhead and fairness for CASA. Figure 7a, b show overhead and fairness for MNIST.

### 4.4 Discussion

IAFB’s superior performance stems from Nash Bargaining optimizing client selection (89% participation on CASA, 90% on MNIST) and Shapley-value incentives ensuring fairness (0.18 Gini on CASA, 0.19 on MNIST), as supported by Figs. 6b, 7b. Compared to Liu et al.’s^11^ reinforcement learning, IAFB reduces convergence time by approximately 14.6% (88 vs. 103 rounds on CASA) and 13.3% (85 vs. 98 rounds on MNIST), as shown in Figs. 4a, 5a. Ma et al.’s^12^ blockchain approach incurs 20% higher overhead (9.0 MB/round vs. IAFB’s 7.5 on CASA, 8.7 vs. 7.8 on MNIST), evident in Figs. 6a, 7a. Fekri et al.’s^13^ privacy methods limit accuracy to 79.2% on CASA and 80.0% on MNIST, while IAFB reaches 91.2% and 90.8%, per Figs. 2a, 3a. Sensitivity analysis shows robustness to learning rate variations (90.5–91.5% accuracy, 0.17–0.20 Gini), and scalability tests validate performance for 50–200 clients (88–92% participation, 90.0–91.2% accuracy). However, Shapley-value computation with Monte Carlo sampling (O($n \cdot k \cdot m$)) increases computational cost for large *n*, a trade-off for fairness. Future work could optimize sampling or explore alternative incentives. IAFB’s efficiency and security make it ideal for IoT smart homes, balancing performance and resource constraints.

## 5. Conclusion

The Incentive-Aware Federated Bargaining framework significantly advances federated learning for IoT smart home environments by addressing critical challenges in client selection, data heterogeneity, security, and participation incentives. Through Nash Bargaining, Shapley-value-based incentives, and decentralized P2P aggregation, IAFB achieves a 28% increase in participation fairness, a 6.5% boost in model accuracy, a 35% reduction in convergence time, and a 39.5% decrease in communication overhead compared to existing FL strategies. Its robust security mechanisms, including AES-GCM encryption, effectively mitigate adversarial threats like Man-in-the-Middle attacks. Future research will focus on integrating lightweight cryptographic techniques, such as homomorphic encryption, to enhance privacy, developing energy-efficient algorithms for resource-constrained devices, and improving scalability through distributed Shapley-value computation. Additionally, incorporating differential privacy and transparent consent mechanisms will further strengthen user trust. In conclusion, IAFB offers a secure, fair, and efficient solution for FL in IoT smart homes, paving the way for scalable, privacy-preserving intelligence in real-world applications.

## Data Availability

The CASA dataset is publicly available at http://casas.wsu.edu/datasets/, and the MNIST dataset is accessible at http://yann.lecun.com/exdb/mnist/ or via torchvision.datasets.MNIST.
